# Differentiating *Plasmodium falciparum *alleles by transforming Cartesian *X,Y *data to polar coordinates

**DOI:** 10.1186/1471-2156-11-57

**Published:** 2010-06-29

**Authors:** Jeana T DaRe, Drew P Kouri, Peter A Zimmerman, Peter J Thomas

**Affiliations:** 1 Center for Global Health and Disease, Case Western Reserve University School of Medicine, Wolstein Research Building, 4-125, Cleveland, Ohio 44106-7286, USA; 2 Department of Mathematics, Case Western Reserve University, 10900 Euclid Avenue, Cleveland, Ohio 44106-7058, USA; 3 Department of Biology, Case Western Reserve University, 10900 Euclid Avenue, Cleveland, Ohio 44106-7058, USA; 4 Department of Cognitive Science, Case Western Reserve University, 10900 Euclid Avenue, Cleveland, Ohio 44106-7058, USA; 5 Department of Neuroscience, Oberlin College, 119 Woodland Street, Oberlin, OH, 44074, USA

## Abstract

**Background:**

Diagnosis of infectious diseases now benefits from advancing technology to perform multiplex analysis of a growing number of variables. These advances enable simultaneous surveillance of markers characterizing species and strain complexity, mutations associated with drug susceptibility, and antigen-based polymorphisms in relation to evaluation of vaccine effectiveness. We have recently developed assays detecting single nucleotide polymorphisms (SNPs) in the *P. falciparum *genome that take advantage of post-PCR ligation detection reaction and fluorescent microsphere labeling strategies. Data from these assays produce a spectrum of outcomes showing that infections result from single to multiple strains. Traditional methods for distinguishing true positive signal from background can cause false positive diagnoses leading to incorrect interpretation of outcomes associated with disease treatment.

**Results:**

Following analysis of *Plasmodium falciparum *dihydrofolate reductase SNPs associated with resistance to a commonly used antimalarial drug, Fansidar (Sulfadoxine/pyrimethamine), and presumably neutral SNPs for parasite strain differentiation, we first evaluated our data after setting a background signal based on the mean plus three standard deviations for known negative control samples. Our analysis of single allelic controls suggested that background for the absent allele increased as the concentration of the target allele increased. To address this problem, we introduced a simple change of variables from customary (*X,Y*) (Cartesian) coordinates to planar polar coordinates (*X *= *r*cos(*θ*), *Y *= *r*sin(*θ*)). Classification of multidimensional fluorescence signals based on histograms of angular and radial data distributions proved more effective than classification based on Cartesian thresholds. Comparison with known diallelic dilution controls suggests that histogram-based classification is effective for major:minor allele concentration ratios as high as 10:1.

**Conclusion:**

We have observed that the diallelic SNP data resulting from analysis of *P. falciparum *mutations is more accurately diagnosed when a simple polar transform of the (*X,Y*) data into (*r,θ*) is used. The development of high through-put methods for genotyping *P. falciparum *SNPs and the refinement of analytical approaches for evaluating these molecular diagnostic results significantly advance the evaluation of parasite population diversity and antimalarial drug resistance.

## Background

Evaluating genotype data of pathogens isolated from a single human host presents an important challenge for molecular diagnostic assays as each human sample can contain varying numbers of parasite strains [1]. Genotype data for many diagnostic technologies are reported across a scale of fluorescence, though further evaluation is necessary to understand how fluorescence units correspond to the number of infecting pathogens. Further complications are encountered if the pathogen is diploid, if target sequences of interest are repeated in single-copy genomes, or if haplotype resolution is a necessary component of diagnosis [2].

We have recently developed a post-PCR, ligase detection reaction - fluorescent microsphere assay (LDR-FMA) to evaluate single nucleotide polymorphisms (SNPs) associated with drug resistance in the notorious malaria parasite, *Plasmodium falciparum *(Pf) [3]. This assay employs Luminex xMAP technology to differentiate between possible alleles at a given locus. The genotypic fluorescence data generated provides semi-quantitative information regarding the relative abundance of haploid parasite strains contained within an individual human patient. In our earlier study, we observed that as the fluorescence signal for single allele positive controls increased with increasing DNA template concentration, fluorescence associated with the allele not present in the sample would increase above background [3].

We approach the problem of accurate genotype identification by transforming bivariate fluorescence data from standard Cartesian (*x,y*) coordinates into polar (*r,θ*) coordinates as described below. In the standard Cartesian representation, the x-axis represents the fluorescence signal in an individual sample associated with allele X (typically the wild-type or drug sensitive allele) and the y-axis represents the fluorescence signal associated with allele Y (typically the mutant or drug resistant allele). This polar transformation takes into account the apparently linear crosstalk interaction responsible for the background signal. A similar kind of bivariate transformation has been applied to genotyping of diploid systems [4-7]. In the diploid case, these methods effectively distinguish between small, discrete numbers of possible diploid copy numbers (e.g. 0, 1 or 2 allelic copies). Our method distinguishes infection by either, both, or neither form of an allele that may be present in any given sample in an effectively continuous range of possible copy numbers.

Accurate classification of background and positive signal is essential in evaluation of infection by any pathogenic species and its various strains. In the context of malaria, isolate differentiation is utilized for studies of parasite population diversity and structure, as well as in identification of drug resistant parasites. Precise estimation of parasite population diversity is essential as this diversity can affect the ability to monitor antimalarial drug efficacy in a region [8]. Genotyping of specific drug resistance loci carries special importance because improper treatment of *P. falciparum *can lead to death of infected patients. Moreover, better identification of drug resistant parasites in endemic populations could be used to revise treatment protocols recommended by Ministries of Health [9] as increasing prevalence of drug resistant parasites has been associated with increased malaria morbidity and mortality [10]. As the arsenal of antimalarial drugs is limited, improved monitoring of drug efficacy and diagnosis of mutations associated with drug resistance is important for programs attempting to control the prevalence of infection by *Plasmodium *species parasites and reduce the potential for mosquito-borne transmission.

## Results and Discussion

### Motivation for the polar coordinate transformation

When diallelic SNPs occur in a population of haploid *P. falciparum *blood-stage parasites, infected individuals may be positive or negative for infection by parasites carrying either allele. With respect to any single SNP locus there are four possible infection "states": X+/Y- (infected by wild-type parasites only), X-/Y+ (infected by mutant parasites only), X+/Y+ (infected by both wild-type and mutant parasites), or X-/Y- (no infection). Three of these states (X+/Y-, X-/Y+, X+/Y+) cannot be distinguished using standard blood smear microscopy because the SNP does not confer observable changes in parasite morphology. The goals of the present study were to distinguish the infection states of individuals in an endemic population and to establish levels of confidence in the diagnostic assessment.

**Figure 1:**
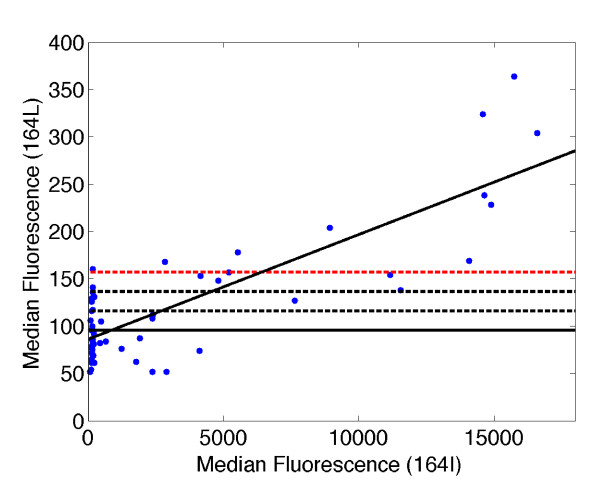
**Detection of the *dhfr *164 SNP alleles: serial dilution of *P. falciparum *3D7 genomic DNA**. Serial dilutions were performed on the laboratory-adapted strain, 3D7 known to carry the 164 I allele in multiple experiments based on two-fold, five-fold, and ten-fold dilutions. The axes of this plot are in Median Fluorescence Intensity (MFI); *x*-axis is MFI for 164 I, *y*-axis is MFI for 164 L. The linear least squares line (solid black; r = 0.82), illustrates how the MFI for 164 L increases as the concentration of 3D7 genomic DNA, and MFI for 164 I increases despite the absence of the 164 L allele in the 3D7 genome. The mean of the background 164 L allele is plotted as a solid black horizontal line; dashed lines designate mean + 1 standard deviation (SD; black), +2 SD (black) and +3 SD (red). Additional experiments focused on the *P. falciparum dhfr *codon 59 SNP of strains Dd2 and 3D7, and dihydropterate synthetase (*dhps*) codon 613 SNP routinely showed this same linear increase in background signal associated with increased concentration of the *dhfr *PCR product.

Unlike genotyping results on single-copy loci from diploid organisms, where allele-specific signals are based on discrete template quantities (0, 1 or 2 allelic copies), allele-specific signals for *P. falciparum *cover a continuous and varying range as a human infection can consist of multiple parasite strains with wide ranging parasitemia levels for each strain. The LDR-FMA procedure produces fluorescence measurements associated with the presence of varying levels of one or two site-specific alleles in a blood sample. Some low-level background signal is observed in control samples containing no DNA. The standard method for establishing the presence of a given allele in a sample is to impose a threshold based on the background signals obtained from a number of blank controls; background signals for alleles of the Pf dihydrofolate reductase gene (*dhfr*) when evaluated in uninfected North American controls ranged from 154 to 322 Median Fluorescence Intensity (MFI) units [3]. A typical threshold would then be set at a fluorescence level of at least two standard deviations above the mean obtained from this ensemble of DNA-negative blanks. In LDR-FMA diagnosis interpretation of true positive from negative fluorescence measurements becomes complicated by background fluorescence signal when, for example, allele "Y" is not present, while allele "X" is present at high levels. Results in Figure 1 illustrate this outcome in a control experiment where LDR-FMA was performed on the *dhfr *SNP occurring at amino acid 164 (sensitive allele = isoleucine [I; codon ATA]; resistant allele = leucine [L; codon TTA]). PCR amplification was performed on a dilution series of the Pf laboratory-adapted strain, 3D7 (*dhfr *164I). The average background signal when no 3D7 genomic DNA was added to the initial PCR was 95.5 MFI units (3xSD = 156.6 MFI units). At higher concentrations of 3D7 genomic DNA the *dhfr *164L signal was consistently above the conventional background value (> 200 MFI units).

**Figure 2:**
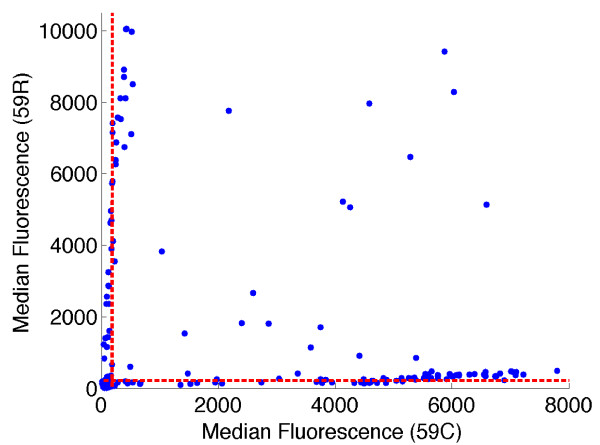
**Detection of the *dhfr *59 SNP alleles from infected Papua New Guinean study participants**. LDR-FMA was performed on 264 clinical samples and the allele-specific MFI data is plotted for *dhfr *59 C/R (*x*-axis is MFI for 59 C, *y*-axis is MFI for 59 R). The mean plus three standard deviations thresholds for each allele are plotted as red dashed lines.

**Figure 3:**
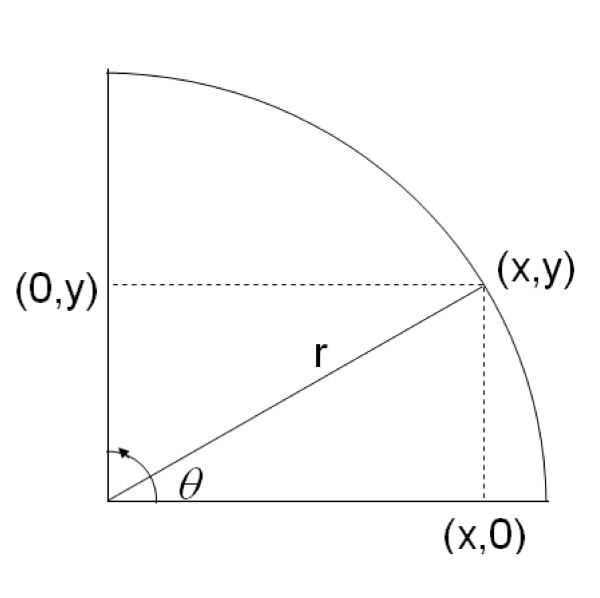
**Polar Transform of Cartesian (*X,Y*) fluorescence data**. The polar transformation is a method that converts Cartesian (*X,Y*) coordinates into their angular (*θ*) and magnitude (*r*) components. Each coordinate (*X *and *Y*) can be written as the product of its magnitude and angle components, i.e. *X*= *r*cos(*θ*) and *Y *= *r*cos(*θ*). From these expressions, we can solve for  and

Because the two sequences differ at only a single base pair, it was not surprising that a small fraction of LDR products representing the 164L allele (not present in 3D7) formed despite sequence mismatch when the concentration of the 164I template increased through PCR amplification. This is particularly true since the mismatched base pairs occur at the end of the oligonucleotide probe sequences and not in middle positions found to be more destabilizing to probe hybridization [11]. In our analyses of field samples from malaria-endemic regions of Papua New Guinea (PNG), we have observed these same background trends in our results. Figure 2 shows an example of bivariate fluorescence data obtained from the LDR-FMA analysis of 264 PNG patients at the *dhfr *59 (C/R) locus. This locus was chosen as both alleles are found in the PNG parasite population, in contrast to the *dhfr *164 locus. Using the threshold determined as three standard deviations above the mean calculated from known negative samples, these results suggest that 68 individuals (25.8%) were infected with mixtures of 59 C and R (Figure 2). When the XY-plot of this data is examined by eye, there is an obvious increase in background levels for both alleles as the median fluorescence increases (Figure 2). Given that similar results were seen in our dilution control experiment (Figure 1), we were concerned that the conventional approach for differentiating positive from background fluorescence was resulting in over-reporting the number of mixed strain infections. Motivated by the possibility that the background signal could be roughly linearly proportional to the concentration of the non-matching sequence, we introduced a transformation of the (*X,Y*) fluorescence data that is compatible with this natural multiplicative structure in the distribution of the data. We introduce polar coordinates (*r,θ*) such that  and  (Figure 3). The magnitude *r *represents the distance in the plane from the point at (0,0) to the point at (x,y), and provides a measure of the overall level of infection. The quantity *θ *represents the angle between the x-axis and ray extending from the point at (0,0) to the point at (x,y). The angle *θ *depends only on the ratio *y/x *of the two fluorescence signals. If the background signal were truly linear, samples with single strain infections would appear in the Cartesian plane along lines with *y/x *ratios equal to a constant. Consequently we sought to determine diagnosis thresholds in terms of the angle *θ *(or equivalently, in terms of the ratio *y/x*) rather than in terms of *x *or *y *alone.

### Histogram based threshold estimation

**Figure 4:**
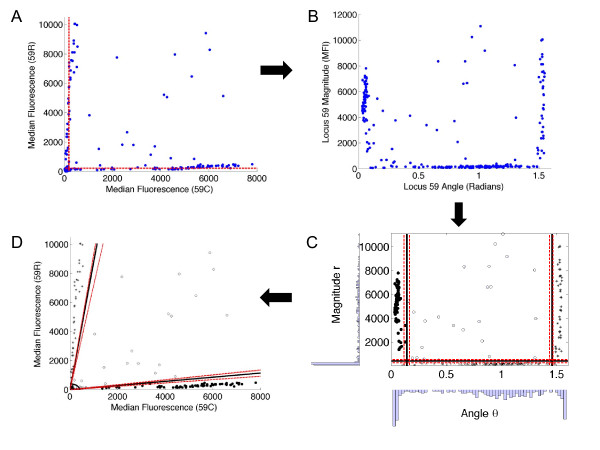
**Rationale for establishing thresholds to differentiate between background and true-positive fluorescence signals**. Panel A: Beginning with raw data, the histogram method transforms the (*X,Y*) data into polar coordinates, (*r,θ*) (Panel B). Panel C: From the polar coordinates, the method generates a histogram of the magnitude, finds the first minimum (magnitude threshold), and removes the negative samples. Then the method generates a histogram of the angular components and determines the first and last minima as the two angular thresholds. Panel D: Finally, the thresholds are transformed back to Cartesian coordinates, where the angular thresholds become lines and the magnitude threshold becomes a quarter circle.

The four possible states of infection observed through the combination of two allele-specific fluorescence signals occupy four regions of the (*X,Y*) plane. Results for individuals with monoallelic infections lie near either the *x*- or *y*-axis, whereas results for mixed strain infections lie somewhere in the upper right region of the plane. Uninfected individuals cluster together near the origin. The classification problem amounts to drawing boundaries between these groups and providing confidence level assessments of the locations of the boundaries. In the (*r,θ*) plane, the transformed fluorescence data again occupy four regions (Figure 4A to 4B). Following the transformation from Cartesian to polar coordinates, the boundaries between each region become parallel to the horizontal or vertical axes, making them straightforward to estimate.

We now introduce a novel algorithmic procedure for determining allele-calling thresholds of individual samples. Our procedure is based on a segmentation technique applied to histograms of data obtained from an entire sample population. In practice we find that the density of bivariate fluorescence measurements obtained empirically typically falls into four distinct clusters corresponding to the four possible states of infection. Our technique exploits the naturally occurring gaps present in histograms produced by such a mixture of differently infected populations.

Our histogram-segmentation analysis of the diallelic population fluorescence data proceeded in two steps. First, we constructed histograms corresponding to the number of data points in the population with magnitudes *r *falling in each of *n*_*r *_equally spaced bins beginning with the minimum magnitude, *r*_- _= min(*r*), and finishing with the maximum magnitude, *r*_+ _= max(*r*). We chose the number of bins as  (this notation indicates that we round the quantity  up to the nearest integer) giving bins of width approximately equal to 100 MFI units (as shown in Figure 4C). The maximum fluorescence levels obtained with Luminex FlexMap microspheres has been observed to be 25,000 MFI, and experimental blanks typically have levels less than 200 MFI. We expected uninfected individuals to have small positive background fluorescence signals clustered together around a central mean (see Methods). Therefore, we determined the first local minimum in the histogram as we increased from the lowest bin, *r*_- _≤ *r *≤ *r*_- _+ 100. Heuristically, the density of points along the r-axis should peak near *r *= *r*_- _and decrease smoothly as we move to successive bins, leaving the uninfected population; the density should reach a minimum before climbing again as one enters the various infected populations. Practically, this method has worked well for data sets ranging in population size from 44 to 667. For very small data sets (*n *≤ 40) this histogram-based method should be used with caution. For further details on implementing the algorithm, see Methods. Sample code written in MATLAB is available from the authors upon request.

Having set a threshold for distinguishing infected from uninfected individuals, we then constructed a histogram of the distribution of angles *θ *of the remaining, infected individuals (Figure 4C). We used 45 equally spaced bins ranging from *θ *= 0 to *θ *= *π*/2 (zero to ninety degrees, in increments of two degrees per bin). We set the threshold for the X-/Y+ population (wild-type infection only) to be the first minimum in the histogram as *θ *increases past the local maximum near *θ *≈ 0. The threshold for the X-/Y+ population (mutant infection only) is the first minimum below the local maximum near  (or 90 degrees). Individuals with wild-type only infections lie scattered around a vertical line at an angle *θ *that is positive (positive slope in the (*X,Y*) plane) but close to zero. Individuals with infections carrying only the mutant allele lie scattered around a vertical line at an angle close to but less than  (or 90°). Individuals with mixed infections were scattered throughout the plane between these two vertical lines, and uninfected individuals lay along the horizontal axis with arbitrary values of *θ*. Once thresholds for distinguishing the four classes of infection/no infection in the polar coordinate plane were established, an inverse polar coordinate transformation returned the data and the diagnosis thresholds to the Cartesian (*X,Y*) plane (Figure 4D). This final recapitulation of the data back into the Cartesian plane illustrates that *the background cutoff lines are not parallel with either the X or Y axes*, as they would have been following previous commonly practiced data analysis strategies (*cf*. Figure 1).

### Confidence Intervals

In order to estimate the probability of classification error following polar transformation of the data, we used bootstrap resampling with replacement to establish percentage confidence interval distributions for the three thresholds (uninfected vs. infected; X+/Y- vs. X+/Y+; X-/Y+ vs. X+/Y+) [12]. With each resampled data set, we used the histogram method to determine thresholds as described above. After running the bootstrap 1,000 to 100,000 times, we determined (1 - *α*) percentile confidence intervals that were placed at the  and  quantiles of the bootstrap estimated thresholds (shown in Figure 4C). We used an increasing series of confidence levels, *α*_1 _<*α*_2 _<...<*α*_*n*_. Each pair of confidence parameters *α*_*j *_<*α*_*j + 1 *_encloses a region of the (*x,y*) plane (or equivalently, the (*r,θ*) plane). A hypothetical point lying in such a region would have a classification confidence between *α*_*j *_and 1-*α*_*j + 1*_. For example, the pair *α*_*j *_= .05 <*α*_*j + 1 *_= .10 would determine a region of the plane corresponding to points with diagnosis confidence exceeding 90%, but not higher than 95%. Table 1 shows the number of points in our sample population lying within a given confidence region. The weighted average of the confidence values gives a lower bound estimate of the average diagnosis error (see Equations 1-2 under Methods). We then used these confidence intervals to obtain upper bounds on the probability that the classification of a randomly chosen member of the population might change upon resampling. If the confidence intervals on the threshold settings were reasonably narrow, then only those individuals whose combined fluorescence measurements located them near one of the thresholds would be in danger of uncertain classification. By placing the thresholds at local minima in the density of sample points we minimized the number of individuals with ambiguous diagnoses. From these estimates, we can determine an upper bound on the probability of misdiagnosis across the entire population (denoted Pr (*error*)). This upper bound on the probability of error in turn gives us a lower bound on the overall confidence of our classifications (as 1-Pr (*error*)), shown in Table 1. For details of this calculation, please see the Methods section.

**Table 1:** Locus-specific uncertainty and confidence intervals

**Confidence Intervals**^**a**^	Dhfr Locus 59	Chr1SNP	Chr7SNP	Chr8SNP	Chr9SNP	Chr13SNP
**0.68**	6	15	1	26	18	23
**0.8**	0	9	15	30	1	3
**0.9**	1	5	8	1	8	6
**0.95**	4	6	1	1	1	0
**0.96**	0	5	0	0	0	2
**0.97**	1	0	3	0	6	0
**0.98**	5	2	0	2	0	6
**0.99**	0	0	0	3	8	0
**0.999**	2	15	14	0	0	5
**> 0.999**^**b**^	245	300	315	231	315	312

**Total Confidence**^**c**^	**0.979**	**0.954**	**0.978**	**0.899**	**0.955**	**0.944**

^a^Confidence intervals for the polar transformation method as determined by bootstrap resampling with replacement. See text for details.

^b^Data points in this category were classified with confidence exceeding 99.9% as estimated via bootstrap resampling.

^c^Total confidence was determined as shown in calculating 1-Pr(error).

The first column of Table 1 shows bootstrap based confidence interval (CI) results for Dhfr locus 59. Out of a total population of 264 samples, six fell within a 68% confidence interval for classification but not within an 80% CI (first row of table). One fell within a 90% CI but was uncertain at the 95% confidence level (third row of table), *et cetera*. The last two rows of Table 1 indicate that two samples were classified with 99.9% confidence, but not better than 99.9%, and the remaining 245 samples were classified with better than 99.9% confidence as estimated by bootstrap resampling. As shown in Table 1 the assignments at each SNP locus based on the polar transform technique had bootstrap classification confidence estimates of 89% or better when averaged across the population.

### Mixed Dilution Control Experiment

**Figure 5:**
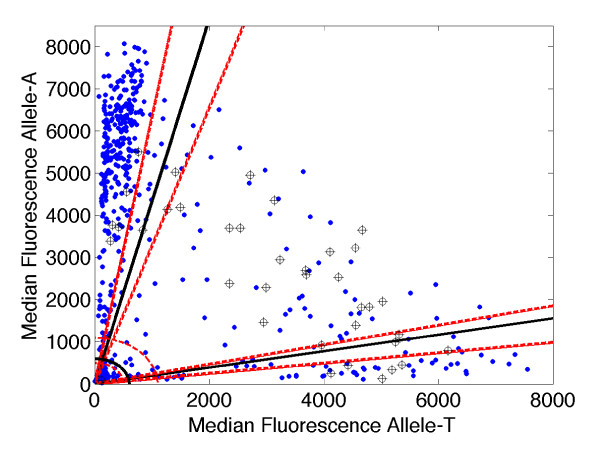
**Control experiment using sample mixtures of known allelic ratios to assess the sensitivity of the histogram segmentation analysis**. The LDR-FMA assay was performed for chr1SNP (CombinedSNP.MAL1.1085, PlasmoDB SNP identifier [13]) on 264 samples collected in PNG (blue dots). The polar coordinate histogram method generated diagnostic thresholds (black solid lines) and 95% confidence intervals (red dotted lines). The LDR-FMA assay was performed separately for the same SNP on 34 mixtures of control strains with known genotypes (HB3, allele-T; K1, allele-A) at DNA concentrations ranging from 1:1 to 99:1 (major allele:minor allele, cross-hair symbols) along with a pure sample of each strain and a blank. Samples correctly called as "mixed" had concentration ratios ranging from 1:1 to as high as 15:1. All samples with major:minor allele ratios of 29:1 or higher were misclassified as "single strain infections". Of samples with major:minor allele ratios of 9:1 or smaller, 95% were called correctly (19 of 20); of those with ratios 10:1 or smaller, over 90% were called correctly (22 of 24).

In order to assess the sensitivity of the histogram segmentation based diagnostic method we used mixtures of laboratory clones (HB3, allele-T; K1, allele-A) with known genotypes to perform control LDR-FMA assays on the chr1SNP (PlasmoDB identifier, CombinedSNP.MAL1.1085 ([13]). We used a total of 34 mixtures with known DNA concentration ratios ranging from 1:1 to 99:1 (major allele:minor allele) along with a pure sample of each allele and a blank (37 samples total). The LDR-FMA assay was also performed on 264 field samples from human subjects collected in PNG. The histogram method was applied to the field data alone in order to generate diagnostic thresholds with 95% confidence intervals (see Figure 5). Subsequently the control samples were called using the thresholds obtained from the field data, and compared with the known DNA concentrations.

The results show that the histogram based method provides reliable results for a range of DNA concentration ratios (analogous to relative parasitemias for two allele types in a given patient) and breaks down once the ratio between major and minor allele concentrations becomes too large. Samples with major:minor allele ratios ranging from 1:1 to 5:1 were called correctly (as mixed infections) 100% of the time (18 out of 18 samples); samples with ratios of 9:1 or 10:1 were called correctly 67% of the time (4 out of 6 samples); both pure samples were called correctly. Mixed samples with ratios of 15:1 were called correctly 50% of the time (1 out of 2 samples) and samples with ratios of 29:1 or higher were always miscalled (as single rather than mixed infections). This performance is expected because of the mismatch binding effect described earlier: when the lower concentration allele is present in too small an amount relative to the major allele, the minor allele disappears below the advancing background signal. Fortunately the method is nevertheless robust over a wide range. In particular, all of the 21 samples called as "mixed" with nominal confidence exceeding 95%, were in fact called correctly. By taking the natural two-dimensional geometry of the data into account, the polar coordinate/histogram segmentation method greatly reduces the risk of false positive diagnoses of mixed infection, without increasing the risk of false negative diagnoses of overall infection. In practice diagnostic assays should therefore include dilutions of allele-specific controls to evaluate potential for misclassification of samples on any given 96-well plate.

### Diagnostic Threshold Variability Across SNPs

We applied the histogram segmentation based diagnostic algorithm to data for six diallelic SNPs (*dhfr *59 and 5 additional SNPs listed in Additional File 1: *Primers and PCR amplification conditions for additional SNPs*) from field samples collected in PNG. Due to a range of factors the angle thresholds (*θ*) for distinguishing mixed from single strain infections varied significantly between SNPs. The X+/Y- versus X+/Y+ threshold (wild-type only versus mixed) ranged from 0.12 to 0.40 radians (7° to 23°) with a mean ± s.d. of 0.22 ± 0.07 radians (12 ± 4)°. The X-/Y+ versus X+/Y+ threshold (mutant only versus mixed) ranged from 1.34 to 1.48 radians (77° to 85°) with a mean ± s.d. of 1.42 ± 0.04 radians (83 ± 2)°. This variability in the natural breakpoint between single and mixed infections may result from SNP-to-SNP variability of practical peak fluorescence, differences in population structure such as relative prevalence of particular SNPs, sequence specific differences in the likelihood of mismatch binding in the LDR assay, among other effects. Because of this variability we cannot fix a single diagnosis threshold in the angle *θ *(or equivalently, in the ratio *y/x*) that will work equally well for all populations and SNPs.

### Comparison of histogram based threshold estimation to conventional Cartesian estimations

Following the finding that our histogram based method provides reliable results for a range of DNA concentration ratios, we can ask how well a method based on Cartesian thresholds would perform in comparison. The standard method employed in malarial SNP genotyping analysis is to set a positive infection threshold based on the distribution of fluorescence observed in an ensemble of blank controls included in the LDR/FMA assay. Typically, this threshold is set at two or three standard deviations above the mean of the blank ensemble fluorescence level.

Table 2 shows the number of individuals assigned to each of four possible states of infection with 95% or higher confidence by the polar transform histogram method, along with the number assigned to the corresponding categories using the standard Cartesian method (mean plus three standard deviations). The final row of the table lists the total number of samples reclassified when the two methods are compared. Even taking into account the number of points classified as "uncertain" by the bootstrap reclassification (an average of 31 samples +/- 15 for six loci examined), the Cartesian method misclassifies a very significant fraction of individuals at each SNP position. This misclassification ranges from 26 to 67% of the samples at each locus, or 76 to 237 samples (Table 2).

**Table 2:** Reclassification of SNP allele calls by use of polar transformation compared to conventional methods

Genotype	Dhfr Locus 59		Chr1SNP		Chr7SNP		Chr8SNP		Chr9SNP		Chr13SNP	
Status	**XY**^**a**^	**Polar**^**b**^	**XY**^**a**^	**Polar**^**b**^	**XY**^**a**^	**Polar**^**b**^	**XY**^**a**^	**Polar**^**b**^	**XY**^**a**^	**Polar**^**b**^	**XY**^**a**^	**Polar**^**b**^
**Not Infected**	127	137	77	104	92	92	70	28	66	95	92	93
**Inf. w/Allele 1**	25^c^	63	19	28	48	60	141	147	4	127	47	108
**Inf. w/Allele 2**	25^d^	37	46	166	102	152	42	27	44	71	77	83
**Mixed Infection**	87	17	215	24	115	28	41	34	243	36	141	41
**Uncertain^e^**	-	10	-	35	-	25	-	58	-	28	-	32

**Total Samples^f^**	**264**		**357**		**357**		**294**		**357**		**357**	
**Reclassified^g^**	**86**		**226**		**96**		**76**		**237**		**126**	

^a^Counts determined based on the conventional threshold of 3× standard deviations above the mean - see Figure 4A.

^b^Counts determined after applying polar transformation method - see Figure 4C.

^c^For Dhfr locus 59, allele 1 is associated with drug sensitivity.

^d^For Dhfr locus 59, allele 2 is associated with drug resistance.

^e^No samples are considered uncertain by conventional (XY) methods, as confidence intervals are not considered.

^f^The total number of samples genotyped at each locus.

^g^The number of samples whose allele calls differed between the XY and polar transformation methods.

This contrast in data analysis is apparent for the classification of infections carrying resistance-associated mutations at *dhfr *codons 59. Whereas 87 samples would be classified by conventional methods at codon 59 as infections including a mixture of parasites carrying both drug sensitive and resistant alleles, only 17 samples were classified to carry this mixed strain infection by the polar transformation method. As mutations in *dhfr *codons 59 are associated with high levels of resistance to drugs targeting the folate biosynthesis pathway [14], misclassification of genotyping results would have significant bearing on the expected susceptibility phenotype for important antimalarial drugs such as sulfadoxine/pyrimethamine (SP, Fansidar) and LAPDAP [15,16].

The misclassification of allele calls by conventional methods at the chr1SNP and chr9SNP are even more apparent than the *dhfr *codon 59 data with 226 and 237 samples respectively reclassified following the use of the polar transformation method (Table 2). This reclassification is caused by high levels of background fluorescence observed for the alleles at these loci as shown in Additional File 2: *Plot of chr1SNP fluorescence data with conventional Cartesian thresholds and thresholds generated by the polar coordinate histogram method*, and Additional File 3: *Plot of chr9SNP fluorescence data with conventional Cartesian thresholds and thresholds generated by the polar coordinate histogram method*. While genotypes at these SNP loci carry no clinical significance, these data indicate that each individual allele specific primer and associated reagents will behave differently in regards to levels of background signal. Accurate allele discrimination for genotyping *P. falciparum *infections therefore requires refined data analysis tools adapted to the intrinsic geometrical structure occurring in population data, such as the histogram based approach introduced here.

### Conclusions

It is to be expected that the data analysis challenges described herein will be observed in other post-PCR genotyping systems, as off-target background signals commonly increase with higher levels of PCR product amplified [17,18]. Accurate discrimination of true positive from background signal when genotyping microbial pathogens by a range of methods, including LDR-FMA and Taq Man, requires novel strategies for data analysis [19,20]. In regards to malaria, isolate differentiation is utilized for studies of parasite population diversity and structure, as well as in identification of drug resistant parasites. The reliability of genotyping methods for strain differentiation can affect the ability to monitor antimalarial drug efficacy and the presence of drug resistance mutations in a parasite population. In the future, the methods described herein can be coupled with expectation maximization approaches to further evaluate multi-locus genotype and haplotype frequencies in parasite populations [21].

## Methods

### Blood samples

Samples evaluated in this study were part of on-going efforts to monitor *Plasmodium *species infections in the Wosera, East Sepik Province, Papua New Guinea (PNG) [22,23]. This region of northern, lowland PNG is holoendemic (parasite rate in one-year old children > 0.75 [24-26]) for malaria and all four human malaria parasite species, *P. falciparum*, *P. vivax*, *P. malariae *and *P. ovale *are observed. Informed consent was obtained from all study participants. This study was approved by the Medical Research Advisory Committee of PNG and by the Institutional Review Board for Human Investigation at University Hospitals of Cleveland, Ohio.

### *P. falciparum *(Pf) laboratory strains

Pf laboratory-adapted strains obtained from the Malaria Research and Reference Reagent Resource (MR4, ATCC Manassas, Virginia) included 3D7 (MRA-102), HB3 (MRA-155), K1 (MRA-159) and VS/1 (MR4-176). *In vitro *growth of Pf was performed as described previously [27]. Thin blood smears were fixed with 100% methanol for 30 seconds, stained with 4% Giemsa for 30 minutes, and examined by microscopy with an oil-immersion objective (100×). Parasitemia was based on the number of infected red blood cells (IRBCs)/total red blood cells (infected plus uninfected RBCs; n = 1,000).

### DNA template preparation

DNA was extracted from whole blood field samples (200 μL) using the QIAamp 96 DNA Blood Kit (Qiagen, Valencia, CA). Genomic DNA was extracted from cultures (200 μL) of laboratory adapted Pf strains using the QIAamp DNA blood mini kit (Qiagen, Valencia, CA).

### PCR amplification of Pf SNPs

All reactions (25 μl) were performed in a buffer containing 3 pmoles of the appropriate upstream and downstream primers, 67 mM Tris-HCl, pH 8.8, 6.7 mM MgSO_4_, 16.6 mM (NH_4_)_2 _SO_4_, 10 mM 2-mercaptoethanol, 100 μ M dATP, dGTP, dCTP, and dTTP, and 2.5 units of thermostable DNA polymerase. Amplification reactions were performed in a Peltier Thermal Cycler, PTC-225 (MJ Research, Watertown, MA). Specific primers and thermocycling conditions used to amplify the Pf dihydrofolate reductase (*dhfr*) target sequence for evaluating polymorphisms associated with pyrimethamine resistance were described in Carnevale et al [3]. Additional, presumably neutral SNPs, chosen irrespective of location or function were utilized for assessing the sensitivity of the histogram segmentation analysis and for examining the diagnostic threshold variability were amplified using the primers and conditions listed in Additional File 1: *Primers and PCR amplification conditions for additional SNPs*. Following PCR amplification, products were loaded on 2% Agarose gels (Amresco, Solon, OH), and electrophoresis was performed in 1× TBE buffer (8.9 mM Tris, 8.9 mM boric acid, 2.0 mM EDTA). The gels were stained for 30 min with SYBR Gold (Molecular Probes, Eugene, OR), diluted 1:10,000 in 1× TBE buffer, and DNA products were visualized on a Storm 860 using ImageQuant software (Molecular Dynamics, Sunnyvale, CA).

### LDR-FMA evaluation of Pf SNPs

**Figure 6:**
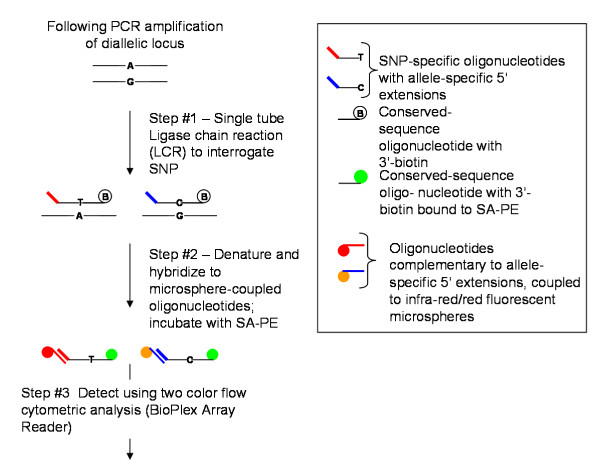
**Overview of post-PCR, LDR-FMA diagnosis of *P. falciparum dhfr *drug resistance-associated SNPS**. Allele-specific oligonucleotides include 5' "TAG" extensions that are 24 nucleotides in length. TAG sequences (n = 100), containing no G residues, have been designed to reduce potential for cross-hybridization with sequence amplified from naturally occurring target sequences. Conserved sequence oligonucleotides are 5'-phosphorylated and 3'-biotinylated. Fluorescent microsphere sets (n = 100) are embedded with varying ratios of red and infra-red fluorochromes to result in unique fluorescent "classification" signatures. Each microsphere set is pre-coupled to unique anti-TAG oligonucleotides specifically complementary to the TAG sequences. Following ligation detection and TAG:anti-TAG hybridization reactions, doubly labeled ligation products are detected and the "reporter" signal (R-phycoerythrin) is classified into allele-specific bins by the BioPlex array reader and BioPlex Manager 3.0 software. Data from these reactions are immediately available in Excel spreadsheets.

The methods and strategies used to perform the LDR-FMA evaluation of Pf mutations in the *dhfr *gene have been previously described in detail [3]. Primers for the LDR-FMA diagnosis of the additional, presumably neutral SNPs chr1SNP, chr7SNP, chr8SNP, chr9SNP, and chr13SNP are listed in Additional File 4: *Ligase detection reaction primers for genotyping additional SNPs*. The following brief description and summary in Figure 6 provide an overview of the three-step, post-PCR, LDR-FMA procedure.

Following PCR amplification of the gene-specific target sequences carrying the locus of interest, products were combined into a multiplex LDR (Step #1) where allele-specific upstream primers ligate to conserved sequence downstream primers. Upstream, allele-specific primers include 5' extensions of unique "TAG" sequences. Downstream, conserved sequence primers are modified by 5' phosphorylation and 3' biotinylation. The 5' ends of the LDR products receive "classification" labeling in a second multiplex (Step #2) reaction where hybridization occurs between the TAG sequences added to the allele-specific primers and anti-TAG (complementary sequence) oligonucleotide probes bound to fluorescent microspheres. Following this hybridization reaction, products are incubated (Step #3) in a solution containing streptavidin-R-phycoerythrin (SA-PE) to allow "reporter" labeling through binding to the 3'-biotin on the conserved sequence primers. Detection of doubly labeled ligation products occurs through dual fluorescence flow cytometry in the BioPlex array reader (Bio-Rad Laboratories, Hercules, CA) and leads to collection of "reporter" signal in unique allele-specific bins. Anti-TAG oligonucleotide probes bound to fluorescent microspheres (2.5 × 10^5 ^beads/mL/US$25) are available from Luminex Corporation (Austin, TX).

Specific LDR primers/probes used for the *dhfr *locus have been previously described [3] and primers/probes for additional SNPs utilized here are listed in Additional Table 2. Individual reactions were performed in a solution (15 μL) containing 20 mM Tris-HCl buffer, pH 7.6, 25 mM potassium acetate, 10 mM magnesium acetate, 1 mM NAD+, 10 mM dithiothrietol, 0.1% Triton X-100, 10 nM (200 fmol) of each LDR probe, 1 μL of each PCR product, and 2 units of Taq DNA ligase (New England Biolabs, Beverly, MA). Reactions were initially heated at 95°C for one minute, followed by 32 thermal cycles at 95°C for 15 seconds (denaturation) and 58.0°C for 2 minutes (annealing/ligation). The multiplex LDR product (5 μL) was then added to 60 μL of hybridization solution (3 M tetramethylammonium chloride [TMAC], 50 mM Tris-HCl, pH 8.0, 3 mM EDTA, pH 8.0, 0.10% sodium dodecyl sulfate) containing 250 Luminex FlexMAP microspheres from each allelic set (total number of alleles = 9). Mixtures were heated to 95°C for 90 seconds and incubated at 37°C for 40 minutes to allow hybridization between SNP-specific LDR products and bead-labeled anti-TAG probes. Following hybridization, 6 μL of streptavidin-R-phycoerythrin (Molecular Probes, Eugene, OR) in TMAC hybridization solution (20 ng/μL) was added to the post-LDR mixture and incubated at 37°C for 40 minutes in Costar-6511M polycarbonate 96-well V-bottom plates (Corning Inc., Corning, NY). Detection of SNP-specific LDR:microsphere-labeled anti-TAG hybrid complexes was performed using a BioPlex array reader (Bio-Rad Laboratories, Hercules, CA); the plate temperature was set to 37°C throughout detection. Fluorescence data, reported as median fluorescence intensity (MFI; range 0 to 25,000), were collected using Bio-Rad software, BioPlex Manager 3.0 (Bio-Rad Laboratories, Hercules, CA).

As a first step in evaluating background signals generated by our LDR-FMA diagnostic assays, we analyzed 70 samples from randomly selected American Red Cross blood donors who had no history of malaria exposure. Through this analysis we observed that median fluorescent intensity (MFI) LDR-FMA signals from these samples were normally distributed. From these results, conventional methods (3× standard deviations above the mean) for establishing thresholds between negative and positive fluorescence were deemed appropriate for comparison against our polar transformation method (Table 2).

### Statistical analyses and graphing

All statistical analyses were performed using MATLAB version 7.7 (R2008a or b) (MathWorks Inc., Boston, MA). After transforming a set of *N *bivariate (*x,y*) fluorescence values into polar (*r,θ*) coordinates and forming the histogram as described above, the "first minimum after the initial maximum" in the magnitude variable was found by applying three criteria. Let *n*(*r*) denote the number of counts in the histogram centered at magnitude *r *and let Δ*r *denote the bin width. (1) The histogram count at the minimum should be less than the counts on either side, or possibly equal to the number on the right, i.e.* n*(*r*-Δ*r*) >*n*(*r*) ≤ *n*(*r *+ Δ*r*). (2) The location of the minimum should exceed the location of the first maximum. The first maximum is required to be a local maximum and to exceed a minimum count requirement, to avoid finding spurious local max/min combinations before the true first population maximum. We used bins of size Δ*r *= 100 MFI units, approximately equal to a single standard deviation of the MFI distribution for uninfected blank samples. We find the following heuristic to be robust in practice: assuming at least 10% of the population falls in the "uninfected" class, we expect approximately 50% of that number to fall within one standard deviation of the uninfected population mean. Therefore we use a threshold for the bin count at the "first maximum" of max(8,*N*/20). (3) We impose a minimum value for the relevant "first minimum after the initial maximum." Assuming bin widths and standard deviation of MFI blank signals of 100 each, we set the bin occupancy threshold equal to twice the number of counts expected to fall between two and three standard deviations from the blank mean, if all *N *samples were actually uninfected. That is, we require(1)

where *erf *denotes the standard error function.

After obtaining a magnitude cutoff (r_*_) for distinguishing infected from uninfected samples, we produce a histogram of the angles *θ *for all *N*_inf _≤ *N *"infected" samples, *i.e*. those with (*r *>r_*_), in 45 bins with a width of 2 degrees ranging from zero to π/2 (ninety degrees). Here let *n*(*θ*) denote the number of counts in the histogram centered at angle *θ *and let Δ*θ *denote the bin width for the angle histogram. Again we apply three criteria to find the wild-type/mixed cutoff (*θ*_*lo*_, close to *θ *= 0) and the mutant/mixed cutoff (*θ*_*hi*_, close to *θ *= π/2). First we find the largest count in the range 0 to *π*/4 (45 degrees) or *π*/4 to *π*/2 (45 to 90 degrees), respectively. (1) Given maximum counts at *θ *= *θ*_0 _(the local maximum close to *θ *= 0) and *θ *= *θ*_90 _(the local maximum close to *θ *= *π*/2) we require *θ*_0 _<*θ*_*lo *_and *θ*_hi _<*θ*_*90*_. (2) We require that the diagnosis thresholds be placed at local minima of the histogram, i.e. *n*(*θ*_*lo *_- Δ*θ*) >*n*(*θ*_*lo*_) ≤ *n*(*θ*_*lo *_+ Δ*θ*) and *n*(*θ*_*hi *_- Δ*θ*) ≥ *n*(*θ*_*hi*_) <*n*(*θ*_*hi *_+ Δ*θ*). (3) In order to be an appropriate local minimum, the count at *θ*_*lo *_or *θ*_*hi *_should not exceed the expected number of counts for the uniform distribution of *N*_inf _samples over 45 bins, or *N*_inf_/45.

### Confidence Intervals

As described in the Results section, we estimated the probability of classification error *via *bootstrap resampling with replacement to establish percentage confidence interval distributions for the three thresholds (uninfected vs. infected; X+/Y- vs. X+/Y+; X-/Y+ vs. X+/Y+) [12]. With each resampled data set, we used the histogram method to determine thresholds. After running the bootstrap 1,000 to 100,000 times, we determined (1 - *α*) percentile confidence intervals that were placed at the  and  quantiles of the bootstrap estimated thresholds (Figure 4C). We used these confidence intervals to obtain upper bounds on the probability that the classification of a randomly chosen member of the population might change upon resampling as follows. Let *ρ*(*r,θ*) denote the density of signal throughout the polar plane, and let *f*(*r,θ*) denote the classification confidence associated with any given point on the polar plane. We would like to approximate the following integral in order to bound the total probability of error by our thresholds (that is, the probability of misclassifying a sample):(2)

for *θ*_- _= min(*θ*) and *θ*_+ _= max(*θ*) (respectively for *r*_- _and *r*_+_). We can rewrite this integral as:(3)

where *x*_*i *_= (*r*_*i*_,*θ*_*i*_) and N is our total sample size. If we choose a subset of confidences, such as {*α*_1_,*α*_2_,...*α*_*n*_} for *α*_*j *_∈ (0,1) then we can obtain an upper bound on the probability of error as the sum of the desired confidences, *α*_*j*_, multiplied by the fraction of the total sample size that lies inside those *α*_*j *_confidence intervals. (For example, a value of *α *= 0.05 corresponds to a 95% confidence.) From these estimates, we obtain a lower bound on the overall confidence in our classifications (see Table 1).

## Abbreviations

SNP: Single nucleotide polymorphism; PNG: Papua New Guinea; LDR: ligase detection reaction; FMA: fluorescent microsphere assay; Pf: *Plasmodium falciparum*; *dhfr*: dihydrofolate reductase; *dhps*: dihydropterate synthetase; *pfcrt*: *P. falciparum *chloroquine resistance transporter; IRBC: infected red blood cell; MFI: median fluorescence intensity; Chr1SNP: PlasmoDB SNP Identifier CombinedSNP.MAL1.1085; Chr7SNP: PlasmoDB SNP Identifier CombinedSNP.MAL7.5506; Chr8SNP: PlasmoDB SNP Identifier CombinedSNP.MAL8.6181; Chr9SNP: PlasmoDB SNP Identifier CombinedSNP.MAL9.4825; Chr13SNP: PlasmoDB SNP Identifier CombinedSNP.MAL13.6337.

## Authors' contributions

PJT developed the mathematical and data analysis framework and advised DPK on implementation. DPK and PJT implemented the algorithms and performed the data analysis. DPK and JD performed the laboratory work and collected the LDR/FMA genotyping data. PAZ developed the PCR-based diagnostic assays. PAZ and PJT jointly supervised the overall project. JD, DPK, PAZ and PJT wrote the manuscript. All authors read and approved the final manuscript.

## Supplementary Material

Additional file 1 **Primers and PCR amplification conditions for additional SNPs**. Primers and conditions for PCR amplification of additional SNPs utilized in assessing the sensitivity of the histogram segmentation analysis and for examining the diagnostic threshold variability. Click here for file

Additional file 2 **Plot of chr1SNP fluorescence data with conventional Cartesian thresholds and thresholds generated by the polar coordinate histogram method**. Samples (n = 357) were genotyped at Chr1SNP using the LDR-FMA method. Allele calls were then made using both the conventional methods (3× standard deviations above the mean of known negative samples) and by the polar coordinate histogram method. Conventional thresholds are indicated by the red dotted line, and the polar thresholds are indicated by the black solid line with the black dotted line showing the 95% confidence intervals. Click here for file

Additional file 3 **Plot of chr9SNP fluorescence data with conventional Cartesian thresholds and thresholds generated by the polar coordinate histogram method**. Samples (n = 357) were genotyped at Chr9SNP using the LDR-FMA method. Allele calls were then made using both the conventional methods (3× standard deviations above the mean of known negative samples) and by the polar coordinate histogram method. Conventional thresholds are indicated by the red dotted line, and the polar thresholds are indicated by the black solid line with the black dotted line showing the 95% confidence intervals. Click here for file

Additional file 4 **Ligation detection reaction primers for genotyping additional SNPs**. Primers for the LDR-FMA diagnosis of additional SNPs utilized in assessing the sensitivity of the histogram segmentation analysis and for examining the diagnostic threshold variability. Click here for file

## References

[B1] Yang S Rothman RE PCR-based diagnostics for infectious diseases: uses limitations, and future applications in acute-care settings Lancet Infect Dis 2004 4 337 348 10.1016/S1473-3099(04)01044-8 15172342 PMC7106425

[B2] Takala SL Smith DL Stine OC Coulibaly D Thera MA Doumbo OK Plowe CV A high-throughput method for quantifying alleles and haplotypes of the malaria vaccine candidate Plasmodium falciparum merozoite surface protein-1 19 kDa Malar J 2006 5 31 10.1186/1475-2875-5-31 16626494 PMC1459863

[B3] Carnevale EP Kouri D DaRe JT McNamara DT Mueller I Zimmerman PA A multiplex ligase detection reaction-fluorescent microsphere assay for simultaneous detection of single nucleotide polymorphisms associated with Plasmodium falciparum drug resistance J Clin Microbiol 2007 45 752 761 10.1128/JCM.01683-06 17121999 PMC1829096

[B4] Moorhead M Hardenbol P Siddiqui F Falkowski M Bruckner C Ireland J Jones HB Jain M Willis TD Faham M Optimal genotype determination in highly multiplexed SNP data Eur J Hum Genet 2006 14 207 215 10.1038/sj.ejhg.5201528 16306880

[B5] Peiffer DA Le JM Steemers FJ Chang W Jenniges T Garcia F Haden K Li J Shaw CA Belmont J High-resolution genomic profiling of chromosomal aberrations using Infinium whole-genome genotyping Genome Res 2006 16 1136 1148 10.1101/gr.5402306 16899659 PMC1557768

[B6] Plagnol V Cooper JD Todd JA Clayton DG A method to address differential bias in genotyping in large-scale association studies PLoS Genet 2007 3 e74 10.1371/journal.pgen.0030074 17511519 PMC1868951

[B7] Teo YY Inouye M Small KS Gwilliam R Deloukas P Kwiatkowski DP Clark TG A genotype calling algorithm for the Illumina BeadArray platform Bioinformatics 2007 23 2741 2746 10.1093/bioinformatics/btm443 17846035 PMC2666488

[B8] Greenhouse B Dokomajilar C Hubbard A Rosenthal PJ Dorsey G Impact of transmission intensity on the accuracy of genotyping to distinguish recrudescence from new infection in antimalarial clinical trials Antimicrob Agents Chemother 2007 51 3096 3103 10.1128/AAC.00159-07 17591848 PMC2043236

[B9] Sibley CH Ringwald P A database of antimalarial drug resistance Malar J 2006 5 48 16774688 10.1186/1475-2875-5-48 PMC1533841

[B10] Trape JF The public health impact of chloroquine resistance in Africa Am J Trop Med Hyg 2001 64 12 17 11425173 10.4269/ajtmh.2001.64.12

[B11] Binder H Preibisch S Kirsten T Base pair interactions and hybridization isotherms of matched and mismatched oligonucleotide probes on microarrays Langmuir 2005 21 9287 9302 10.1021/la051231s 16171364

[B12] Wasserman L All of Statistics: A Concise Course in Statistical Inference 2004 1 New York: Springer

[B13] Aurrecoechea C Brestelli J Brunk BP Dommer J Fischer S Gajria B Gao X Gingle A Grant G Harb OS PlasmoDB: a functional genomic database for malaria parasites Nucleic Acids Res 2009 37 D539 543 10.1093/nar/gkn814 18957442 PMC2686598

[B14] Sibley CH Hyde JE Sims PF Plowe CV Kublin JG Mberu EK Cowman AF Winstanley PA Watkins WM Nzila AM Pyrimethamine-sulfadoxine resistance in Plasmodium falciparum: what next? Trends Parasitol 2001 17 582 588 10.1016/S1471-4922(01)02085-2 11756042

[B15] Nzila-Mounda A Mberu EK Sibley CH Plowe CV Winstanley PA Watkins WM Kenyan Plasmodium falciparum field isolates: correlation between pyrimethamine and chlorcycloguanil activity in vitro and point mutations in the dihydrofolate reductase domain Antimicrob Agents Chemother 1998 42 164 169 9449279 10.1128/aac.42.1.164 PMC105474

[B16] Wilairatana P Kyle DE Looareesuwan S Chinwongprom K Amradee S White NJ Watkins WM Poor efficacy of antimalarial biguanide-dapsone combinations in the treatment of acute uncomplicated, falciparum malaria in Thailand Ann Trop Med Parasitol 1997 91 125 132 10.1080/00034989761454 9307653

[B17] Liu H Li S Wang Z Ji M Nie L He N High-throughput SNP genotyping based on solid-phase PCR on magnetic nanoparticles with dual-color hybridization J Biotechnol 2007 131 217 222 10.1016/j.jbiotec.2007.06.023 17719116

[B18] Takatsu K Yokomaku T Kurata S Kanagawa T A FRET-based analysis of SNPs without fluorescent probes Nucleic Acids Res 2004 32 e156 10.1093/nar/gnh155 15534363 PMC528829

[B19] Troyer RM Lalonde MS Fraundorf E Demers KR Kyeyune F Mugyenyi P Syed A Whalen CC Bajunirwe F Arts EJ A radiolabeled oligonucleotide ligation assay demonstrates the high frequency of nevirapine resistance mutations in HIV type 1 quasispecies of NVP-treated and untreated mother-infant pairs from Uganda AIDS Res Hum Retroviruses 2008 24 235 250 10.1089/aid.2007.0138 18284323

[B20] Daniels R Volkman SK Milner DA Mahesh N Neafsey DE Park DJ Rosen D Angelino E Sabeti PC Wirth DF Wiegand RC A general SNP-based molecular barcode for Plasmodium falciparum identification and tracking Malar J 2008 7 223 10.1186/1475-2875-7-223 18959790 PMC2584654

[B21] Li X Foulkes AS Yucel RM Rich SM An expectation maximization approach to estimate malaria haplotype frequencies in multiply infected children Stat Appl Genet Mol Biol 2007 6 Article33 18052916 10.2202/1544-6115.1321

[B22] Genton B al-Yaman F Beck HP Hii J Mellor S Narara A Gibson N Smith T Alpers MP The epidemiology of malaria in the Wosera area East Sepik Province Papua New Guinea in preparation for vaccine trials. I. Malariometric indices and immunity Ann Trop Med Parasitol 1995 89 359 376 7487223 10.1080/00034983.1995.11812965

[B23] Kasehagen LJ Mueller I McNamara DT Bockarie MJ Kiniboro B Rare L Lorry K Kastens W Reeder JC Kazura JW Zimmerman PA Changing patterns of Plasmodium blood-stage infections in the Wosera region of Papua New Guinea monitored by light microscopy and high throughput PCR diagnosis Am J Trop Med Hyg 2006 75 588 596 17038678 PMC3728901

[B24] Lysenko A Semashko I Geography of malaria: A medico-geographic profile of an ancient disease 1968 Moscow: Academy of Sciences USSR

[B25] Metselaar D Thiel PV Classification of Malaria Tropical and Geographical Medicine 1959 11 157 161

[B26] Snow RW Guerra CA Noor AM Myint HY Hay SI The global distribution of clinical episodes of Plasmodium falciparum malaria Nature 2005 434 214 217 10.1038/nature03342 15759000 PMC3128492

[B27] McNamara DT Kasehagen LJ Grimberg BT Cole-Tobian J Collins WE Zimmerman PA Diagnosing infection levels of four human malaria parasite species by a polymerase chain reaction/ligase detection reaction fluorescent microsphere-based assay Am J Trop Med Hyg 2006 74 413 421 16525099 PMC3728833

