# Supplementary material for: Differentiating Plasmodium falciparum alleles by transforming Cartesian X,Y data to polar coordinates
Source: BMC Genet. 2010 Jun 29;11:57. doi: 10.1186/1471-2156-11-57 (PMC2912781; doi:10.1186/1471-2156-11-57)
Supplement: Additional file 1 — Primers and PCR amplification conditions for additional SNPs. Primers and conditions for PCR amplification of additional SNPs utilized in assessing the sensitivity of the histogram segmentation analysis and for examining the diagnostic threshold variability. [file 1471-2156-11-57-S1.PDF]

Additional File 1: Primers and PCR amplification conditions for additional SNPs

PlasmoDB SNP identifier	Abbreviated SNP Name	Primer sequences	Amplification conditions
CombinedSNP.MAL1.1085	Chr1SNP	Up 5'- cgataagtagttgtgaattatg -3'<br>Dn 5'- atatgaggtctctatattagga -3'	95°C 2 min (1x), 95°C 30 sec, 56°C 30 sec, 60°C 1 min (35x), 60°C 4 min (1x)
CombinedSNP.MAL7.5506	Chr7SNP	Up 5'- catatgatgcatcacattcag -3'<br>Dn 5'- ctatcattgtctccatttca -3'	95°C 2 min (1x), 95°C 30 sec, 56°C 30 sec, 60°C 1 min (35x), 60°C 4 min (1x)
CombinedSNP.MAL8.6181	Chr8SNP	Up 5'- ctctttctttccatctaagc -3'<br>Dn 5'- ggtgaatttagaggattgga -3'	95°C 2 min (1x), 95°C 30 sec, 56°C 30 sec, 65°C 1 min (35x), 65°C 4 min (1x)
CombinedSNP.MAL9.4825	Chr9SNP	Up 5'- ttgataaatgatgatacgaaaaa -3'<br>Dn 5'- gaactgcagaaaataccatc -3'	95°C 2 min (1x), 95°C 30 sec, 60°C 30 sec, 65°C 1 min (35x), 65°C 4 min (1x)
CombinedSNP.MAL13.6337	Chr13SNP	Up 5'- tattgagtttattttttgtttatt -3'<br>Dn 5'- gttagtaggtactatttctata -3'	95°C 2 min (1x), 95°C 30 sec, 56°C 30 sec, 60°C 1 min (35x), 60°C 4 min (1x)
