# Supplementary material for: Differentiating Plasmodium falciparum alleles by transforming Cartesian X,Y data to polar coordinates
Source: BMC Genet. 2010 Jun 29;11:57. doi: 10.1186/1471-2156-11-57 (PMC2912781; doi:10.1186/1471-2156-11-57)

Additional File 3: Plot of chr9SNP fluorescence data with conventional Cartesian thresholds and thresholds generated by the polar coordinate histogram method.

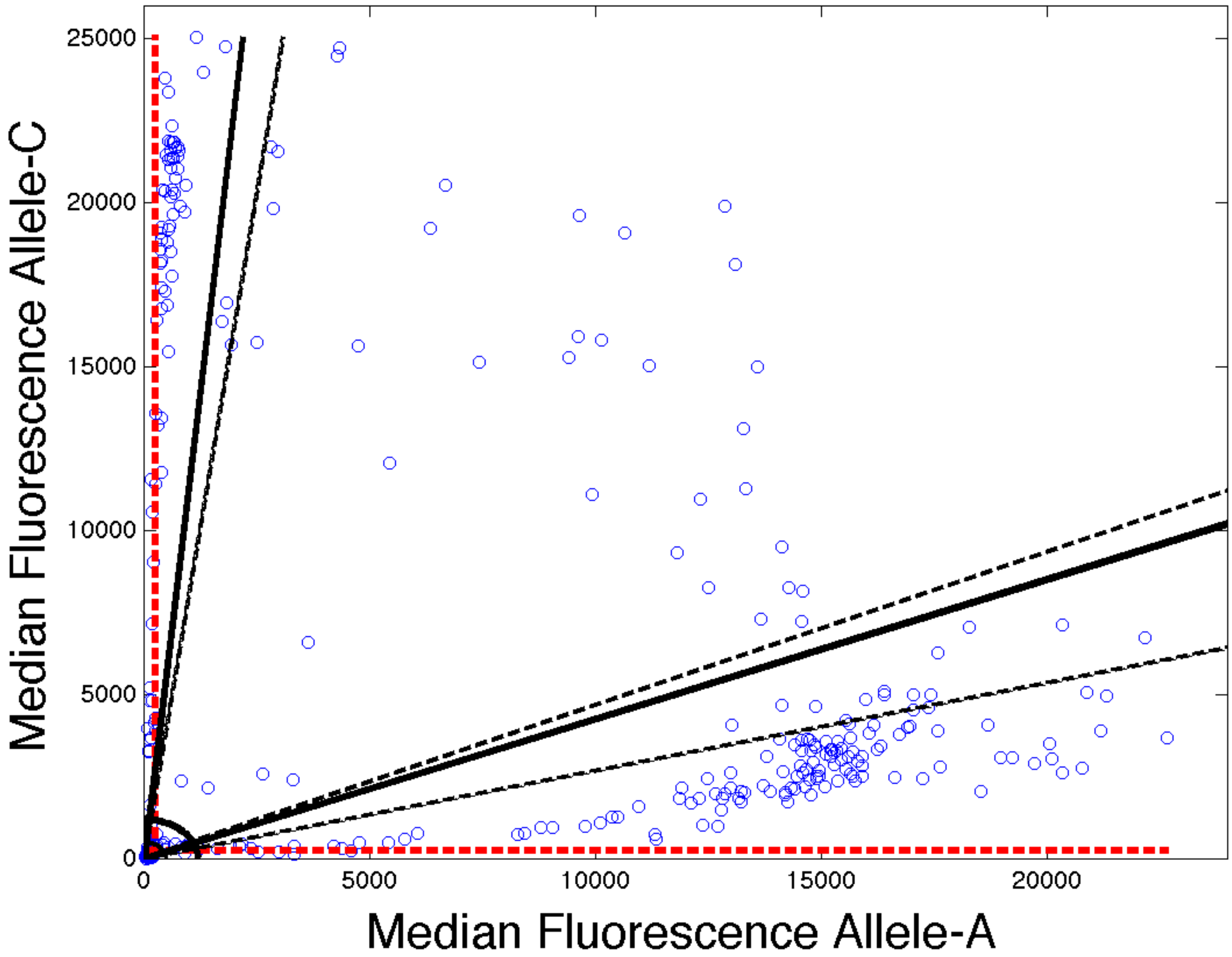

Supplement: Additional file 3 — Plot of chr9SNP fluorescence data with conventional Cartesian thresholds and thresholds generated by the polar coordinate histogram method. Samples (n = 357) were genotyped at Chr9SNP using the LDR-FMA method. Allele calls were then made using both the conventional methods (3× standard deviations above the mean of known negative samples) and by the polar coordinate histogram method. Conventional thresholds are indicated by the red dotted line, and the polar thresholds are indicated by the black solid line with the black dotted line showing the 95% confidence intervals. [file 1471-2156-11-57-S3.PDF]
