# Supplementary material for: Differentiating Plasmodium falciparum alleles by transforming Cartesian X,Y data to polar coordinates
Source: BMC Genet. 2010 Jun 29;11:57. doi: 10.1186/1471-2156-11-57 (PMC2912781; doi:10.1186/1471-2156-11-57)
Supplement: Additional file 4 — Ligation detection reaction primers for genotyping additional SNPs. Primers for the LDR-FMA diagnosis of additional SNPs utilized in assessing the sensitivity of the histogram segmentation analysis and for examining the diagnostic threshold variability. [file 1471-2156-11-57-S4.PDF]

Additional File 4: Ligase detection reaction primers for genotyping additional SNPs

PlasmoDB SNP identifier - allele	Primer Sequence[a]	FlexMap^{TM} microsphere
CombinedSNP.MAL1.1085 - A	5'- CTTTTCATCAATAATCTTACCTTTagcattaaatgaacaaatatcaa -3'	65
CombinedSNP.MAL1.1085 - T	5'- TCAAAATCTCAAATACTCAAATCAagcattaaatgaacaaatatcat -3'	18
CombinedSNP.MAL1.1085 - Common	5'/Phos/ tatttttaaattggatttttaaatc -3' Biotin	
CombinedSNP.MAL7.5506 - T	5'- TACATTACCAATAATCTTCAAATCttcatgttcacaatttgataaat -3'	4
CombinedSNP.MAL7.5506 - A	5'- TAATCTTCTATATCAACATCTTACttcatgttcacaatttgataaaa -3'	9
CombinedSNP.MAL7.5506 - Common	5'/Phos/ ataaactattcttattattattgtc -3' Biotin	
CombinedSNP.MAL8.6181 - G	5'- CAATAAACTATACTTCTTCACTAAtccttccaaccataattttag -3'	13
CombinedSNP.MAL8.6181 - A	5'- CAATTCATTTACCAATTTACCAATtccttccaaccataattttaa -3'	7
CombinedSNP.MAL8.6181 - Common	5'/Phos/ aagttgccattcttcttctg -3' Biotin	
CombinedSNP.MAL9.4825 - C	5'- ATACCAATAATCCAATTCATATCAatgtgatgcagatgccac -3'	70
CombinedSNP.MAL9.4825 - A	5'- TTACTTCACTTTCTATTTACAATCgatgtgatgcagatgccaa -3'	88
CombinedSNP.MAL9.4825 - Common	5'/Phos/ atgtaccgaagaagattcag -3' Biotin	
CombinedSNP.MAL13.6337 - G	5'- CAATTCAAATCACAATAATCAATCgtattgtgttgagcttaatag -3'	5
CombinedSNP.MAL13.6337 - T	5'- CTTTAATCCTTTATCACTTTATCAgtattgtgttgagcttaatat -3'	17
CombinedSNP.MAL13.6337 - Common	5'/Phos/ ttcactaatactttttttacc -3' Biotin	

a Nucleotides in upper case letters (24 bases) represent the TAG sequence added to the 5’ end of each allele-specific LDR primer.
